# An Intersectional Approach to Target Neural Circuits With Cell- and Projection-Type Specificity: Validation in the Mesolimbic Dopamine System

**DOI:** 10.3389/fnmol.2019.00049

**Published:** 2019-02-28

**Authors:** Nefeli Kakava-Georgiadou, Maria M. Zwartkruis, Clara Bullich-Vilarrubias, Mieneke C. M. Luijendijk, Keith M. Garner, Geoffrey van der Plasse, Roger A. H. Adan

**Affiliations:** ^1^Division of Neuroscience, Department of Translational Neuroscience, Brain Center Rudolf Magnus, University Medical Center Utrecht, Utrecht, Netherlands; ^2^Master’s Program Neuroscience and Cognition, Utrecht University, Utrecht, Netherlands; ^3^Institute of Neuroscience and Physiology, The Sahlgrenska Academy at the University of Gothenburg, Gothenburg, Sweden

**Keywords:** VTA, dopamine, DREADD, chemogenetics, Cav2, canine, CNO, Flp

## Abstract

Development of tools to manipulate activity of specific neurons is important for dissecting the function of neural circuits. Viral vectors and conditional transgenic animal lines that target recombinases to specific cells facilitate the successful manipulation and recording of specific subsets of neurons. So far, it has been possible to target neuronal subtypes within a certain brain region based on transcriptional control regions from a gene selectively expressed in those cells or based upon its projections. Nevertheless, there are only a few tools available that combine this and target a neuronal subtype within a projection. We tested a viral vector system, consisting of a canine adenovirus type 2 expressing a Cre-dependent Flp recombinase (CavFlexFlp) and an adeno-associated viral (AAV) vector expressing a Flp-dependent cDNA, which targets neurons in a subtype- and projection-specific manner. As proof of principle we targeted expression of a Designer Receptor Exclusively Activated by Designer Drugs (DREADD) to the dopamine neurons of the mesolimbic projection, which allows the transient activation of neurons by the ligand Clozapine-N-Oxide (CNO). We validated that the system specifically targets dopamine neurons and that chemogenetic activation of these neurons induces an increase in locomotor activity. We thus validated a valuable tool that allows *in vivo* neuronal activation in a projection- and subtype-specific manner.

## Introduction

Investigating the roles of specific subsets of neurons in behavior is a major challenge in neuroscience. To this end, several genetic tools have been developed for manipulation of neuronal activity *in vivo*. Common tools for transient activation or inactivation of neurons are opto- and chemogenetics. Optogenetics utilizes expression of light-sensitive ion channels which are selectively activated by light at a precise millisecond scale, causing neuronal depolarization, thereby mimicking the physiological function of neurons through action potentials (Fenno et al., 2011). Chemogenetics, on the other hand, allows for a longer-lasting but still transient manipulation of neuronal activity, via expression of Designer Receptors Exclusively Activated by Designer Drugs (DREADDs). Mutated human muscarinic receptors are the most commonly used DREADDs and are exclusively activated by designer drugs such as Clozapine-N-Oxide (CNO), which – in most cases – are administered systemically to the animals (Roth, 2016). Chemogenetic tools are used to transiently activate or inhibit neurons and determine their role in behavior or mimic physiological or pathological situations of general neuronal hyper- or hypo-activity.

The further development of genetic tools to more efficiently and specifically target neural specific cells will help neuroscientists to gain knowledge about the role of specific neurons in behavior and disease.

The most common strategy to express chemogenetic tools in the central nervous system (CNS) is to use viral vectors, such as the adeno-associated virus (AAV) as well as viral vectors that allow retrograde transfer, such as canine adenovirus type 2 (Cav2) (Davidson and Breakefield, 2003; Betley and Sternson, 2011; Gholizadeh et al., 2013; Junyent and Kremer, 2015). The development of the Cre/lox system has facilitated the successful delivery and expression of these tools in either a subtype or projection-specific manner. For example, the combination of Cre-dependent DREADDs with the retrograde viral vector Cav2-Cre injected in an output region permits projection- (but not subtype-) specific circuit manipulation (Boender et al., 2014; Augur et al., 2016). Instead, the delivery of Cre-dependent hM3D(Gq) DREADD in Cre-driver mouse or rat lines allows for the subtype- (but not projection-) specific activation of neurons with CNO (Krashes et al., 2011; Atasoy et al., 2012; Boekhoudt et al., 2016). Since DREADDs are also expressed in neuronal terminals, by using a subtype-specific system and locally applying CNO with a cannula in the output region, it is possible to activate neuronal subtypes in a subtype- and projection-specific manner (Stachniak et al., 2014; Verharen et al., 2018). Nevertheless, this technique is invasive to the animal – since it requires the placement of the cannula – and is time-consuming due to the time it takes to deliver CNO compared to an i.p. injection. Finally, even though the location and spread of viral expression can be assessed post-mortem, it is not possible to assess these parameters for drug delivery and there is also a possibility of off-target activation of passing fibers of neurons that belong to a different projection.

We aimed to further develop and validate a strategy that facilitates projection- and subtype- specific delivery of DREADD in neuronal subtypes. As a model system we targeted ventral tegmental area (VTA) dopamine neurons that project to the Nucleus Accumbens (NAc). We created a DREADD viral vector which we use in a double conditional system in order to achieve projection- and subtype-specific activation. To this end, we used TH::Cre rats, in which Cre recombinase is expressed in Tyrosine Hydroxylase neurons (a marker for dopamine neurons in the VTA). In these rats we injected: (1) CavFlexFlp in the NAc, which delivers the recombinase flippase (Flp) retrogradely in a Cre-dependent manner, thereby expressing Flp in all TH^+^ neurons that project to NAc (Schwarz et al., 2015) and (2) a new Flp-dependent Gq-coupled DREADD [frt-DREADDq] in the VTA, which brings expression of this DREADD in dopamine cells in the VTA>NAc projection. We assessed the ability of the system to specifically target mesolimbic dopamine neurons and tested the efficacy of the system to manipulate behavior.

## Materials and Methods

### Animals

Adult TH::Cre (^+/-^) and (^-/-^) rats on a Long-Evans background [provided by K. Deisseroth (Witten et al., 2011)] and adult Pvalb-2A-FlpO-D (^+/-^) and (^-/-^) mice (022730, Jackson Laboratories) on a C57Bl/6J background were used. Male and female animals that were used for immunohistochemical assessment only were housed socially and kept under a normal 12:12 h light-dark cycle with lights off at 19:00. For behavior experiments male rats were housed individually and kept under a reverse 12:12 h light-dark cycle with lights off at 07:00. All animals were kept at room temperature (21 ± 2°C) and 40–60% of humidity conditions. They were provided with chow and water *ad libitum*. All experiments were approved by the Animal Ethics Committee of Utrecht University and conducted in agreement with Dutch laws (Wet op de Dierproeven, 1996; revised 2014) and European regulations (Guideline 86/609/EEC; Directive 2010/63/EU).

### Plasmid Construction and Viruses

Plasmids pAAV-EF1a-DIO-hM3D(Gq)-mCherry and pAAV-hSyn-dF-HA-KORD-IRES-mCitrine (pAAV-frt-KORD-mCitrine), gifts from Bryan Roth (Addgene plasmids #50460 and #65417), were digested with restriction enzymes AscI and NheI and hM3D(Gq):mCherry insert and pAAV-hSyn-dF backbone were ligated to generate plasmid pAAV-hSyn-frt-hM3D(Gq):mCherry. PCR with forward primer 5’-GCTAGCATGGTGAGCAAGGGCGAG-3’ (Additional AscI restriction site on 5’ end) and reverse primer 5’-GGCGCGCCTTACTTGTACAG-3’ was performed on pAAV-EF1a-DIO-hM3D(Gq)-mCherry, the product was ligated into pGEMT.easy (Promega) and after restriction digest with AscI and NheI the mCherry insert was ligated into backbone pAAV-hSyn-dF, to generate plasmid pAAV-hSyn-frt-mCherry. The sequence of the constructs was confirmed with Sanger sequencing. Plasmid pAAV-hSyn-DIO-mCarMyc_f-ChetaHA_r (pAAV-mCAR) was provided by Li et al. (2018).

Serotype 5 AAV viruses were generated as described earlier (Backer, 2010), except that the vectors pAAV-hSyn-frt-hM3D(Gq):mCherry, pAAV-hSyn-frt-mCherry, pAAV-frt-KORD-mCitrine, and pAAV-mCAR were co-transfected with the pDP5 plasmid (Grimm et al., 2003) at a molar ratio of 1:1, resulting in AAV vectors rAAV5-hSyn-frt-hM3D(Gq):mCherry (frt-DREADDq), rAAV5-hSyn-frt-mCherry (frt-mCherry), rAAV5-frt-KORD-IRES-mCitirine (frt-KORD), rAAV5-mCAR (AAV-mCAR). Serotype 8 virus was generated by co-transfecting plasmids pAAV-hSyn-frt-hM3D(Gq):mCherry, pAR-8 (provided by M. Sena Esteves) and pXX-680 [provided by R. Samulski (Grieger et al., 2016)], at a molar ratio of 1:1:1 to create vector rAAV8-hSyn-frt- hM3D(Gq):mCherry. The plasmid pAAV-EF1a-Cre (gift from Karl Deisseroth, addgene plasmid # 55636) was co-transfected with the plasmid pAAV2-retro helper (gift from Alla Karpova and David Schaffer, addgene plasmid # 81070) and plasmid pXX-680 at a molar ratio of 1:1:1 to create vector retro-AAV2-Cre (Retro-Cre). Titer of viruses was determined using real-time PCR (qPCR) with primers binding on the wPRE element or mCherry. CAV-FLEx^loxP^-Flp (CavFlexFlp) and Cav2-Cre were purchased from IGMM, Montpellier, France and rAAV5-hSyn-DIO-hM3D(Gq):mCherry (lox-DREADDq) was purchased from UNC Vector Core and Addgene (Addgene viral prep # 44361-AAV5).

### Stereotaxic Surgeries

All viruses were injected on the same day, except for rAAV5-mCAR, which was injected 2 weeks prior to the rest of the viruses. Rats were anesthetized with an intramuscular injection of hypnorm (0.315 mg/kg fentanyl and 10 mg/kg fluanisone, Janssen Pharmaceutica, Beerse, Belgium). Mice were anesthetized with ketamine (75 mg/kg, Narketan) and medetomidine (1 mg/kg, Seda Start). Animals were placed on a stereotaxic apparatus (David Kopf Instruments, Tujunga, United States or Configuration Stereotaxic, SGL Manip 18° – 68U801, UNO, Netherlands) and a small incision was made along the midline of the skull. Viruses were injected using a 34G stainless steel needle connected to 10 μl Hamilton syringe at a rate of 0.2 μL/min. In PV-FlpO mice, 1 μL of rAAV5-hSyn-frt-hM3D(Gq):mCherry (2 × 10^12^g.c./ml) was injected into the ventral hippocampus (-3.50 mm anteroposterior (AP), ± 2.90 mm mediolateral (ML) from Bregma, and -4.70 mm dorsoventral (DV) from the skull, no angle). In rats, 0.3–1 μl of CAV-FLEx^loxP^-Flp (2.8 × 10^12^g.c./ml) or Cav2-Cre or retroCre (2 × 10^12^g.c./ml) or saline (0,9% NaCl) were bilaterally injected into the NAc (+1.20 mm anteroposterior (AP), ± 2.80 mm mediolateral (ML) from Bregma, and -7.50 mm dorsoventral (DV) from the skull, at an angle of 10°) and rAAV5-mCAR or rAAV5-hSyn-frt-KORD-IRES-mCitrine or rAAV5-hSyn-DIO-hM3D(Gq):mCherry (2 × 10^12^g.c./ml) or rAAV5-hSyn-frt-hM3D(Gq):mCherry or rAAV5-hSyn-frt-mCherry (2.5-5 × 10^12^g.c./ml) into the VTA (-5.60 mm AP, ± 1.30 mm ML from Bregma, and -8.20 mm DV from the skull, at an angle of 5°). After injection, the needle was maintained at its injection position for 15 min. After surgery, carprofen was administered for pain relief (5 mg/kg per day for 3 days, subcutaneous, s.c.) and saline (1 ml/100 g in rats and 0.4 ml/10 g in mice, once, s.c.).

### Histology

**FIGURE 1:**
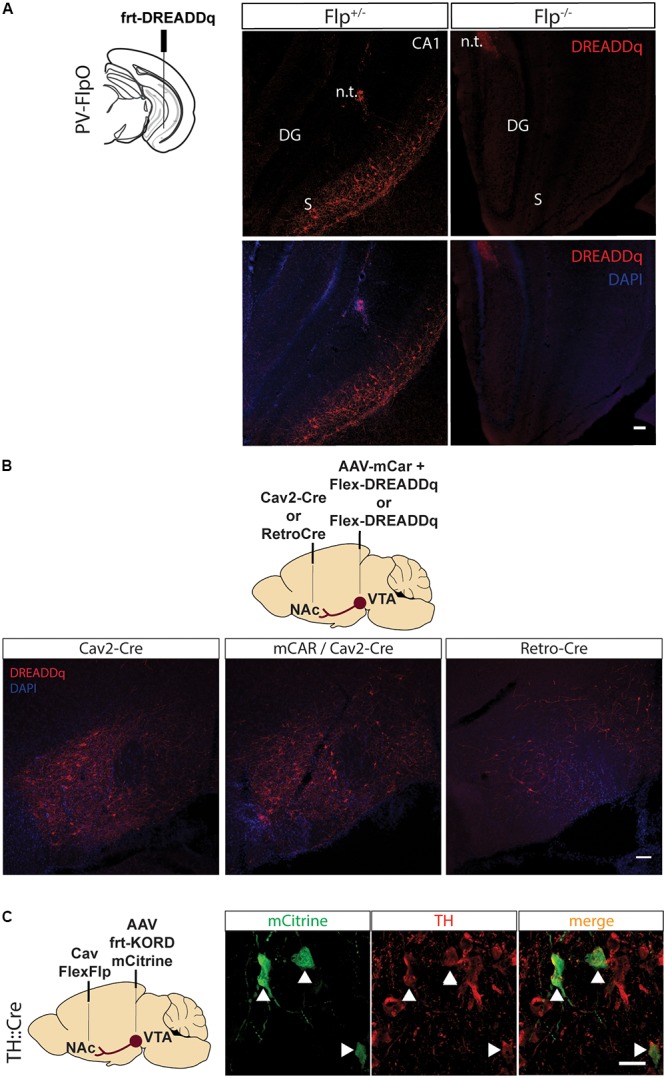
Pilot testing of viral vectors. **(A)** PV-FlpO^+/-^ and ^-/-^ mice were injected in the ventral hippocampus with frt-DREADDq. Immunohistochemistry against mCherry (fused to DREADDq) showed expression of DREADDq in sections from ventral hippocampus of PV-FlpO^+/-^ but not of PV-FlpO^-/-^ littermates. **(B)** Rats were injected in the NAc and VTA with a combination of Cav2-Cre and lox-DREADDq (Cav2-Cre) or Cav2-Cre and lox-DREADDq with AAV-mCAR (mCAR/Cav2-Cre) or Retro-Cre and lox-DREADDq (Retro-Cre) respectively. Immunohistochemical detection of mCherry (fused to DREADDq) showed no difference in expression levels between Cav2-Cre and mCAR/Cav2-Cre, whereas Retro-Cre brought lower levels of mCherry expression. **(C)** TH::Cre rats were injected with CavFlexFlp and frt-KORD-mCitrine in the NAc and VTA, respectively, so that VTA>NAc dopamine neurons would be targeted. Immunohistochemistry against mCitrine (green) and TH (red) showed co-localization of the mCitrine^+^ with TH^+^ neurons (white arrows). n.t., needle tract; CA1, field CA1 of the hippocampus; DG, Dentate Gyrus; S, Subicculum.

**FIGURE 2:**
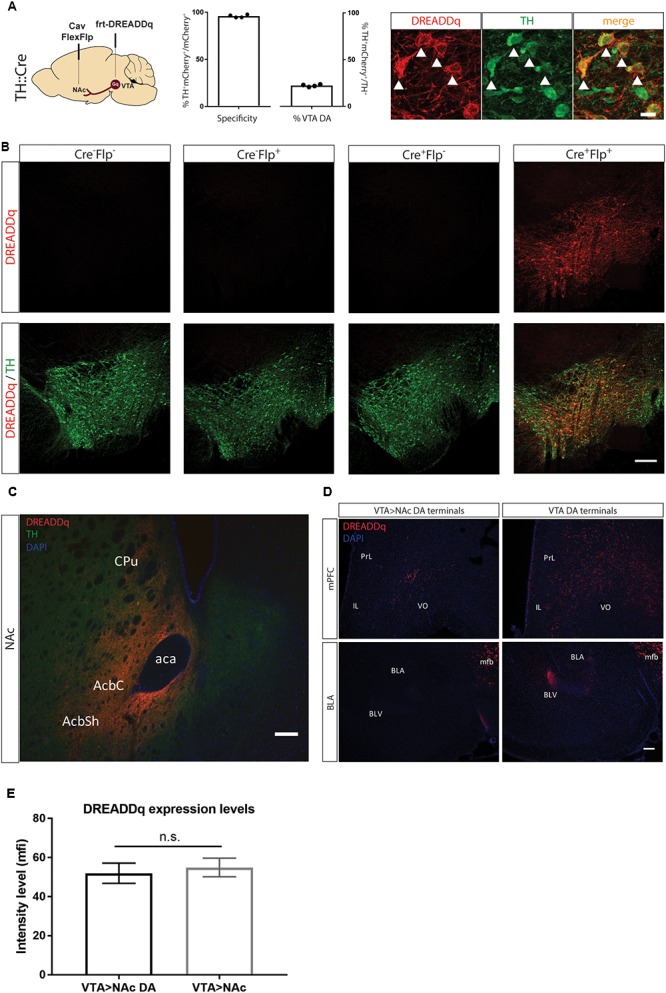
Targeting VTA>NAc dopamine neurons with DREADDq. **(A)** TH::Cre^+/-^ rats were injected in the NAc and VTA with CavFlexFlp and frt-DREADDq, respectively. Co-staining for mCherry (fused to DREADDq, red) and TH (green), showed co-localization (white arrows), with 22,2% ± 0,6 of the total VTA TH^+^ neurons targeted (% mCherry^+^TH^+^ / total TH^+^) and specificity of 95,9% ± 0,8 (% mCherry^+^TH^+^ / total mCherry^+^). **(B)** TH::Cre^-/-^ rats were injected with frt-DREADDq in the VTA (Cre^-^Flp^-^) or with CavFlexFlp in the NAc and frt-DREADDq in the VTA (Cre^-^Flp^+^); TH:Cre^+/-^ rats were injected with frt-DREADDq in the VTA (Cre^+^Flp^-^) or with CavFlexFlp in the NAc and frt-DREADDq in the VTA (Cre^+^Flp^+^); Immunohistochemical detection of mCherry (fused to DREADDq) showed no expression of DREADDq in Cre^-^Flp^-^, Cre^-^Flp^+^ and Cre^+^Flp^-^ rats, whereas in Cre^+^Flp^+^ rats DREADDDq was expressed in the VTA. **(C)** Fibers positive for mCherry (red) were detected in the NAc shell and core, where TH^+^ fibers are also detected (green) in TH::Cre^+/-^ rats injected in the NAc and VTA with CavFlexFlp and frt-DREADDq, respectively. **(D)** In TH::Cre^+/-^ rats injected in the VTA with lox-DREADDq (VTA DA) fibers positive for mCherry (fused to DREADDq) were detected in the mPFC and the BLA (panels on the right), whereas in TH::Cre^+/-^ rats injected in the NAc and VTA with CavFlexFlp and frt-DREADDq, respectively few or no mCherry^+^ fibers were detected in the mPFC and the BLA, respectively (panels on the left). **(E)** Expression levels of DREADDq were measured by quantification of mCherry intensity (as mean fluorescent intensity – mfi) in TH::Cre^+/-^ rats injected in the NAc and VTA with CavFlexFlp and frt-DREADDq, respectively (VTA>NAc DA) and TH::Cre^-/-^ rats injected in the NAc and VTA with Cav2-Cre and lox-DREADDq, respectively (VTA>NAc). No difference was found between VTA>NAc DA and VTA>NAc targeting (unpaired *t*-test; *t*(25) = 0,4093, *p* = 0.6858); Data represented as mean ± SEM; ns, not significant; Cpu, Caudate putamen; AcbC, Accumbens nucleus, Core; AcbSh, Accumbens nucleus, Shell; aca, anterior commissure; PrL, Prelimbic cortex; IL, Infralimbic cortex; VO, Ventral Orbital cortex; BLA, Basolateral Amygdaloid nucleus, anterior; BLV, Basolateral Amygdaloid nucleus, ventral; mfb, medial forebrain bundle.

Mice used in Figure 1A were perfused 2 weeks after surgery. Rats used in Figure 1B,C, 2A,B,D were perfused 7, 4, 5, 7, and 7 weeks after the last surgery, respectively. Rats used in Figure 2E were from various experiments and they were perfused 7 to 17 weeks after surgery, timepoints balanced between groups. Animals were sacrificed with a sodium pentobarbital overdose (200 mg/mL, Euthanimal, Alfasan BV, Netherlands). Animals were perfused with ice-cold 1× phosphate buffered saline (PBS) pH 7.3, followed by ice-cold 4% paraformaldehyde (PFA) in 1×PBS pH 7.3. Brains were removed and incubated overnight in 4% PFA, then transferred consecutively to 20% for 1 day and 30% for 2 days sucrose solution in 1×PBS. After the brains had sunk in the sucrose solution, they were snap-frozen by isopentane immersion and stored at -80°C. Coronal sections were sliced at 40 μm thickness in a cryostat (Leica, Germany).

### Immunohistochemistry

Sections were blocked with 10% normal goat serum (NGS), 1% Triton X-100 in 1×PBS, followed by overnight incubation with primary antibodies (1:500 mouse anti-TH, MAB318, Milipore; 1:750 rabbit anti-dsRed,632496, Clontech; 1:500 rabbit anti-TH, Ab152, Milipore; 1:1000 chicken anti-GFP, ab13970, Abcam) in 3% NGS, 0,25% Triton X-100 in 1× PBS. Sections were incubated with the secondary antibodies (1:500 goat anti-mouse 488,ab150113, Abcam; 1:500 goat anti-rabbit 568,ab175471, Abcam; 1:500 goat anti-chicken 488, ab150169, Abcam) for 1 h at room temperature and DAPI (1:1000 in PBS 1×) for 15’. Between all steps sections were washed three times for 5–10 min in PBS 1×. Sections were then mounted on microscope glasses, let to dry and covered with Fluorsave Reagent (345789, Calbiochem).

### Imaging and Image Analysis

For quantifications of specificity and efficiency, pictures were taken at 20× magnification at a confocal microscope (Olympus Fluoview FV1000, Olympus, Japan). Cells were manually counted using the Cell Counter plugin of ImageJ. For intensity analysis, 10× magnification pictures were taken with an epi-fluorescent microscope (Zeiss Scope A1, ZEISS, Germany). Intensity was measured on ImageJ and background fluorescence was subtracted. All images within a figure part (e.g., in 1C) were taken with the same exposure and brightness and contrast were altered in the same levels.

### Drugs

Clozapine-N-oxide (CNO; kindly provided by Bryan Roth and NIMH or purchased from AK scientific Cat. No. AMTA056) was dissolved in sterile saline (0.9% NaCl). All injections were given intra-peritoneally (i.p.) at 1 ml per kg body weight, and CNO solution was kept at 4°C in between injections, for a maximum of 1 week.

### Locomotor Activity

At 4 weeks after surgery rats were acclimatized for 1 h to PhenoTyper^®^ 9000 cages (Noldus IT, Wageningen, Netherlands), 43 × 43 × 90 cm, equipped with infrared video cameras in the top to monitor locomotor activity, and to intraperitoneal (i.p.) injections with sterile saline (0.9% NaCl) for at least two times before starting with testing. Behavioral testing started at least 5 weeks after surgery, to allow virus expression to reach its maximum. On the day of the testing, at 10:00 or 13:00 (counterbalanced between groups), rats were habituated to the PhenoTyper cages for 30 min, injected i.p. with CNO or vehicle (saline) counterbalanced between groups and locomotor activity was recorded for 2 h after injections. The test was repeated 48 h after the first test day and rats that previously received CNO, now received saline and vice versa. Locomotor activity was recorded at 25 samples per second, and was analyzed with EthoVision XT11.5 (Noldus IT, Wageningen, Netherlands). Movement tracks of the animals’ center point were smoothed by locally weighted scatterplot smoothing.

### Statistical Analyses

Statistical analyses were performed with GraphPad Prism 7.0 and IBM SPSS version 13. Data was checked for normality and non-parametric tests were performed when data did not follow a Gaussian distribution. Rats with unilateral or no expression were excluded from analysis of locomotor activity.

## Results

In order to achieve projection- and subtype-specific expression of transgenes, we utilized a double conditional system comprising of a Cre-driver rat expressing Cre in tyrosine hydroxylase positive cells (TH::Cre), the retrograde virus CAV-FLEx^loxP^-Flp (CavFlexFlp) in the Nucleus Accumbens (NAc), which infects neurons at their terminals and expresses Flp only in the presence of Cre, and an AAV virus expressing a transgene only in the presence of Flp in the ventral tegmental area (VTA). In order to test this system’s specificity and efficiency, we applied it to dopaminergic neurons of the mesolimbic projection and assessed histological and behavioral parameters.

### Pilot Testing of Viral Vectors

As a first step to test the system, we performed a series of pilot experiments to assess the specificity of Flp-dependent transgene expression and to find the most efficient way to target the VTA>NAc projection.

First, we created an rAAV5-frt-hM3D(Gq):mCherry (frt-DREADDq), a Flp-dependent DREADD receptor which allows transient activation of neurons when CNO is administered. This virus was expressed when injected into the ventral hippocampus of parvalbumin (PV)-FlpO^+/-^ mice but not in wild-type (PV-FlpO^-/-^) littermates (Figure 1A), therefore confirming that it is expressed only in the presence of Flp recombinase.

In an effort to find the most efficient viral vector system to target VTA>NAc projection neurons, we tested novel tools that are known to increase retrograde transfer. Canine adenovirus 2 (Cav2) vectors are robustly used in neuroscience to target projections. Therefore, we injected Cav2-Cre in the NAc and Cre-dependent lox-DREADDq in the VTA (Cav2-Cre). In a second group of rats we aimed to increase Cav uptake by inducing expression of coxsackievirus and adenovirus receptor (CAR) in the neuronal terminals of the VTA>NAc projection, by injecting the VTA with AAV-mCAR 2 weeks prior the injections of Cav2-Cre in the NAc and lox-DREADDq in the VTA of rats (mCar/Cav2-Cre) (Li et al., 2018). Finally, in a third group of rats we injected Retro-Cre (a virus expressing Cre packaged in the retro-AAV2 variant) (Tervo et al., 2016) in the NAc and lox-DREADDq in the VTA (Retro-Cre). We compared the expression of DREADDq between these three groups. Overall, we observed equal levels of expression of DREADDq in the mCar/Cav2-Cre and Cav2-Cre groups and much lower levels in the Retro-Cre group (Figure 1B). Therefore, using a Cav2 was the most efficient way in our hands to target the VTA>NAc projection in rats.

CavFlexFlp retrogradely delivers Flp in a Cre-dependent manner (Schwarz et al., 2015). In order to assess the specificity of this vector, we injected CavFlexFlp in the NAc and a previously established frt-KORD-mCitrine (Vardy et al., 2015) into the VTA of TH::Cre rats. VTA TH^+^ (dopamine) neurons stained for mCitrine with a specificity of 91,15% ± 0,95 (mean ± SEM, *n* = 2, Figure 1C).

We thus demonstrated the specificity of each of the vectors which we aimed to combine in subsequent experiments.

### Targeting VTA>NAc Dopamine Neurons With DREADDq

We next injected CavFlexFlp in the NAc and rAAV5- or rAAV8-frt-DREADDq in the VTA of TH::Cre rats (*n* = 2/group). We did not observe differences in expression between the two serotypes, suggesting similar infection efficacy of the two different viral coats and therefore combined the results. 22,2% ± 0,6 of the total VTA TH^+^ neurons were targeted with a specificity of 95,9% ± 0,8 (Figure 2A). In all subsequent experiments serotype 5 was used.

To further confirm the specificity of the viral vector system, we determined expression of mCherry in the absence of either or both of the recombinases Cre (by using a non-transgenic littermate) and Flp (by injecting saline instead of CavFlexFlp), the presence of which is essential for expression. We found no expression in all three control conditions (Figure 2B).

Next, we explored whether there is expression of DREADDq in the projection terminal sites of mesolimbic dopamine neurons in the NAc. We found robust expression of mCherry (fused to DREADDq) in the NAc core and shell (Figure 2C) as well as along the needle tract in the dorsomedial part of the striatum (not shown).

Moreover, we examined two more major output sites of VTA dopamine neurons: the medial prefrontal cortex (mPFC) and basolateral amygdala (BLA) in order to assess if VTA>NAc dopamine neurons make collaterals to these regions (Lammel et al., 2008; Yetnikoff et al., 2014). We found fewer or no DREADDq^+^ fibers in the mPFC and BLA, respectively, in TH::Cre rats injected with frt-DREADDq and CavFlexFlp in the VTA and the NAc, respectively (VTA>NAc DA) compared to TH::Cre rats injected with DIO-Gq in the VTA (VTA DA) (Figure 2D).

Considering that dopamine neurons form a subpopulation of the VTA>NAc projection, we expected that fewer neurons would express DREADDq when targeting the VTA>NAc DA projection compared to targeting the VTA>NAc projection. In a pilot experiment, we found that in TH::Cre rats injected with CavFlexFlp in the NAc and frt-DREADDq in the VTA (VTA>NAc DA), the total number of DREADDq^+^ neurons in the VTA was 645 ± 51 (mean ± SEM, *n* = 2). In Long-Evans rats injected with Cav2-Cre in the NAc and lox-DREADDq in the VTA (VTA>NAc), 661,5 ± 74,25 (mean ± SEM; *n* = 2) DREADDq^+^ neurons were counted in the VTA. To further investigate whether there is a difference in expression levels, we measured DREADDq expression in larger groups of animals, by quantifying intensity of mCherry, which is fused to DREADDq. We found no difference in DREADDq expression levels between VTA>NAc DA and VTA DA groups (unpaired *t*-test; *t*(25) = 0,4093, *p* = 0.6858, Figure 2E). Therefore, we concluded that targeting neurons at a subtype- and projection-specific level was as efficient as targeting neurons at a projection-specific level.

### Activation of VTA>NAc DA Neurons Increases Locomotor Activity

Next, we aimed to investigate the efficiency of the system to manipulate behavior. Chemogenetic activation of VTA DA neurons or VTA>NAc neurons leads to locomotor hyperactivity (Boender et al., 2014; Boekhoudt et al., 2016). Therefore, we used locomotor activity as an outcome measure in order to assess the efficiency of the system to drive behavior.

We injected CavFlexFlp and frt-DREADDq into the NAc and VTA of TH::Cre^+/-^ rats, respectively (*n* = 13, Gq group). As controls we used TH::Cre^-/-^ rats (*n* = 5, Cre^-^ group) as well as TH::Cre^+/-^ rats injected instead with frt-mCherry (*n* = 4, Gq^-^ group). After at least 5 weeks, we measured the distanced moved in 2 h after CNO and vehicle i.p. administration.

There was no effect of CNO administration on locomotor activity in neither the Cre^-^ group (Friedman test; χ^2^(5) = 0.4, *p* = 0.9537) or the Gq^-^ group (Friedman test; χ^2^(4) = 4.5, *p* = 0.1250). Therefore, we merged the two groups in order to increase the power in subsequent analyses (*n* = 9, Ctl group).

**FIGURE 3:**
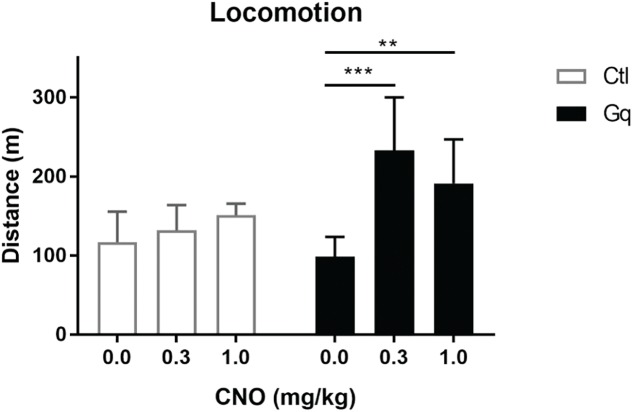
Activation of VTA>NAc DA neurons increases locomotor activity. Gq rats were TH::Cre^+/-^ rats injected in the NAc and VTA with CavFlexFlp and frt-DREADDq, respectively (*n* = 13); Ctl rats were either TH::Cre^+/-^ rats injected in the NAc and VTA with CavFlexFlp and frt-mCherry, respectively, or TH::Cre^-/-^ rats injected in the NAc and VTA with CavFlexFlp and frt-DREADDq, respectively (total *n* = 9); CNO administration did not increase locomotor activity in Ctl rats (Friedman test; χ^2^(9) = 1.556, *p* = 0.5690), whereas CNO significantly increased locomotor activity in Gq rats (Friedman test; χ^2^(13) = 0.333, *p* = 0.0002; Dunn’s multiple comparisons *post hoc* test: Saline vs. CNO 0.3 mg/kg *p* = 0.0004; Saline vs. CNO 1.0 mg/kg *p* = 0.0017); Data represented as median with interquartile range; ^∗∗^*P* < 0.01, ^∗∗∗^*P* < 0.001.

Activation of mesolimbic dopamine neurons with CNO (at 0.3 and 1.0 mg/kg) significantly increased distance moved in 2 h after injection in the Gq group (Friedman test; χ^2^(13) = 0.333, *p* = 0.0002; Dunn’s multiple comparisons *post hoc* test: Saline vs. CNO 0.3 mg/kg *p* = 0.0004; Saline vs. CNO 1.0 mg/kg *p* = 0.0017), whereas CNO had no effect in the Ctl group (Friedman test; (χ^2^(9) = 1.556, *p* = 0.5690) (Figure 3).

## Discussion

We here assessed a novel tool that targets and allows to transiently activate neurons in a subtype- and projection-specific manner. We demonstrate that the combination of CavFlexFlp with AAV-frt-DREADDq specifically targets mesolimbic dopamine neurons in TH::Cre rats. We also show that CNO increases locomotor activity, showing that sufficient numbers of mesolimbic dopamine neurons were brought under chemogenetic control using this strategy.

In particular, we created a Flp-dependent hM3D(Gq) DREADD which is only expressed in the presence of Flp recombinase (Figure 1A). Next, we assessed which is the most efficient viral vector to target the VTA>NAc projection.

Cav2 viruses use coxsackievirus and adenovirus receptors (CAR) in neuronal terminals to infect neurons. Therefore, enhancing expression of CAR may enhance Cav2 tropism, resulting in higher expression of DREADDq. To this end, we targeted the VTA>NAc projection with Cav2-Cre in the NAc and a combination of AAV-mCAR and lox-DREADDq in the VTA (Li et al., 2018). We did not observe differences in expression of DREADDq after targeting mCAR in the VTA compared to just injecting Cav2-Cre in the NAc, suggesting that VTA dopamine neurons are sensitive enough to be infected by Cav and infection is not further increased by expressing CAR. Another tool for retrograde delivery is a retro-AAV2 carrying Cre recombinase (Tervo et al., 2016). We injected Retro-Cre in the NAc and lox-DREADDq in the VTA and observed that Retro-Cre was less efficient than the previous tools to target the VTA to NAc projection in rats (Figure 1B). This data shows that Cav2 is the most efficient way to target the VTA>NAc projection in rats, without the need for a prior injection with AAV-mCAR.

Next, we expressed DREADDq in the dopamine neurons of the VTA>NAc projection by targeting the VTA with frt-DREADDq and the NAc with retrograde Cre-dependent Flp (CavFlexFlp) in TH::Cre rats, and showed that DREADDq is only expressed when both Cre and Flp are present (Figure 2B). We also show that terminals in the NAc express DREADDq using this strategy (Figure 2C). We found no expression of DREADDq in the BLA, showing that the dopamine neurons targeted in this study do not make collaterals to this region. Moreover, we found very low expression in the mPFC which might be collaterals, but we cannot exclude that this was caused by spread of CavFlexFlp (Figure 2D). Even though this is not a major finding, we confirmed previous studies that mostly show that VTA>NAc dopamine projection neurons do not make collaterals to PFC or BLA (Lammel et al., 2008; Yetnikoff et al., 2014). Nevertheless, we cannot exclude the possibility that there might be collaterals to the regions investigated or other regions in the brain (Aransay et al., 2015). However, it is beyond the aims of this study to investigate this further.

In our hands, we did not find a difference in DREADDq expression levels between VTA>NAc and VTA>NAc DA targeting, despite the fact that dopamine neurons constitute around 80% of the VTA>NAc projection (Figure 2E). This suggests that efficacy to target specifically dopamine neurons in the VTA>NAc projection is not compromised when two recombinases (Cre and Flp) rather than one (Cre) need to be active to achieve proper recombination.

Dopamine-specific expression of DREADDq in the midbrain, as well as VTA>NAc expression of DREADDq drives CNO-induced locomotor activity (Wang et al., 2013; Boender et al., 2014; Boekhoudt et al., 2016). While our work was in preparation, it was shown that chemogenetic deactivation of VTA>NAc DA neurons in TH-Cre mice, after injection of CavFlexFlp in the NAc and Flp-dependent hM4D(Gi) in the VTA, results in reduction in cocaine-induced hyperlocomotion (Runegaard et al., 2018). In line with this, we found that chemogenetic activation of VTA>NAc DA neurons in rats induced a hyperactive phenotype. In particular, administration of CNO increased locomotor activity around twofold compared to Saline in the Gq (VTA>NAc DA) group but not in the control group (Figure 3). The twofold increase in locomotor activity is at a similar magnitude with the increase observed in Boender et al. (2014), when projection-specific (VTA>NAc) activation was induced. Nevertheless, Boekhoudt et al. (2016) observed a sevenfold increase when activating either VTA DA or VTA>NAc neurons at the same CNO doses as we used. One explanation for why we did not observe such an increase is that during VTA DA activation more populations of DA neurons contribute to the hyperactive phenotype. Secondly, the additional activation of GABA neurons, which constitute around 20% of the VTA>NAc projection, might contribute to the higher increase in locomotor activity when VTA>NAc neurons are chemogenetically activated. Another explanation might be that Boekhoudt et al. (2016) tested locomotion in the homecage, in which the baseline locomotion was already at lower levels when testing, whereas we tested the effect of CNO activation in a more novel environment, with 30 min habituation prior to injection. Finally, the lower magnitude of increase that we observed might be due to lower efficiency of the system to target neurons. We injected frt-DREADDq in the VTA at lower titers (2.5–5 × 10^12^ g.c./mL) compared to the titers of lox-DREADDq injected by Boekhoudt et al. (2016) (6.4–8 × 10^12^ g.c./mL). The lower amount of genomic copies might explain a lower efficiency of expression. In the future, in order to target more neurons, higher titers of frt-DREADDq could be applied but with caution, because specificity could be compromised.

In conclusion, we tested and validated cell-type and projection- specific systems to transiently activate neurons and study behavior. We showed that combining CavFlexFlp and AAV-frt-DREADDq in TH::Cre transgenic rats specifically targets VTA>NAc dopamine neurons and that activation by CNO increases locomotor activity. The usefulness of this strategy remains to be determined for other VTA dopamine projections. This system has great potential to be applied in a variety of Cre-transgenic lines to target, record, and manipulate the activity of specific cell-types in different projections. Increasing the efficiency of the system might be achieved by increasing titers and using more efficient tools for retrograde delivery.

## Data Availability

The datasets generated for this study are available on request to the corresponding author.

## Author Contributions

NK-G, GvdP, and RA conceptualized the manuscript. NK-G, MZ, CB-V, ML, and KG performed the experiments. NK did analysis of the data. NK and RA prepared the original draft. All authors reviewed the manuscript.

## Conflict of Interest Statement

The authors declare that the research was conducted in the absence of any commercial or financial relationships that could be construed as a potential conflict of interest.
